# Artificial intelligence-assisted development of *in situ* forming nanoparticles for arthritis therapy via intra-articular delivery

**DOI:** 10.1080/10717544.2022.2069882

**Published:** 2022-05-09

**Authors:** Ahmed S. Yacoub, Hussein O. Ammar, Magdy Ibrahim, Suzan M. Mansour, Nada M. El Hoffy

**Affiliations:** a Department of Pharmaceutics and Pharmaceutical Technology, Faculty of Pharmacy, Future University in Egypt, Cairo, Egypt; b Department of Pharmaceutics and Industrial Pharmacy, Faculty of Pharmacy, Cairo University, Cairo, Egypt; c Department of Pharmacology and Toxicology, Faculty of Pharmacy, Cairo University, Cairo, Egypt; d Department of Pharmacology, Toxicology and Biochemistry, Faculty of Pharmacy, Future University in Egypt, Cairo, Egypt

**Keywords:** *In-situ* forming nanoparticles, rheumatic arthritis, piroxicam, intra-articular, targeted drug delivery

## Abstract

Intra-articular (IA) injection is grasping much interest due to the poor drug bioavailability at the targeted site of action which minimizes the effect of the orally administered moiety. Based on the integral role of non-steroidal anti-inflammatory drugs (NSAIDs) in the treatment of Rheumatoid Arthritis (RA), much effort is exerted to develop novel localized drug delivery systems to increase their bioavailability and minimize their side effects. Artificial intelligence (AI) is acquiring an increasing role in the design of experiments being an effective tool for saving both time and resources. Hence, the aim of this work was to develop, characterize and optimize targeted *in-situ* forming nano particles (ISNs) for IA delivery of piroxicam using Design^®^ Expert as an AI-based application where a 3^3^ full factorial experimental design was adopted. Morphological investigation, injectability, rheological studies, Fourier Transform Infrared Radiation (FTIR) as well as biological, histopathological, and biochemical examinations were performed to evaluate the optimized-ISNs. The optimized formulation, exhibiting a nano-sized particle size with a dense core, showed significant improvement in the histopathological findings compared to both the oral solution and the placebo. Additionally, the once-a-week IA administration of the optimized-ISNs proved a significant reduction in the protein expression of both STAT-3 and RANKL and the levels of anti-CCP and MCP-1 by almost 54 and 73%, respectively, coupled with a marked decline in the content of IL-17, MMP-3, NF-κB and TNF-α as compared to the positive control. In conclusion, the use of ISNs for intra-articular injection has demonstrated their effectiveness in piroxicam delivery for RA treatment.

## 1. Introduction

The development of novel drug discovery technologies like combinatorial chemistry, genetic engineering, and high-throughput screening results in higher therapeutic potential in many drug candidates which suffer from bad oral absorption or a short biological half-life. Moreover, these advancements in drug discoveries have drawn significant attention on the development of innovative techniques to deliver them efficiently and effectively. A pioneer of such approaches is the parenteral controlled drug delivery systems. These systems can, after a single administration, keep the drug within the desired therapeutic range for a considerably long time via different routes of administration and supporting various dosage forms like emulsions, oil solutions, liposomes, implants, micelles, and microparticles. The administration of such systems results in the formation of an injection site depot that serves as a drug reservoir. Compared to conventional oral route of administration, they offer several advantages including increased bioavailability, prolonged release, constant drug plasma concentration and localized drug delivery (Luan, 2006). The nature of the vehicle, physical and chemical characteristics of the drug, as well as the interaction of drug with vehicle and tissue fluid determine the rate of absorption of the drug and, consequently, the duration of its therapeutic activity (Saravanakumar et al., 2019).

Intra-articular (IA) drug delivery represents a significant breakthrough of such parenteral systems especially in the treatment of chronic conditions as rheumatoid arthritis where the drug is being injected directly into the affected area and released over a longer time period (Butoescu et al., 2009). Various techniques are available to control the drug release in such systems as implants or microparticles which form drug-biodegradable polymer composites (Siepmann & Siepmann, 2006). Many problems have been reported with the preparation and drug loading procedures of these systems, including increased process temperature, poor homogeneity of content (specifically with low-dose drugs) and the ongoing need for invasive administration in case of implants. In addition, the preparation of biodegradable implants is complex and includes multi-step procedures with close monitoring of formulation parameters which consequently has an impact on scale-up and cost (Wang et al., 2003; Schwendeman et al., 2014; Ahmed, 2015).

Focus has been drawn to the formulation of injectable biodegradable and biocompatible polymeric particles, of both natural and synthetic origins, such as microspheres, microcapsules, nano-capsules and nanospheres. These formulations were used to avoid inconvenience accompanied with surgical procedure for insertion of bulky implants with optimum size ranging from 250 µm to 125 µm (Lengyel et al., 2019). These novel drug delivery systems have been shown to reduce IA drug clearance and increase the mean residence time compared to conventional dosage forms. A major drawback of such systems is the significantly high burst release and high viscosity of polymer solutions that cause injection problems (Tiwari et al., 2012). Extensively searching alternatives, novel *in situ* forming microparticle (ISM) formulations have been developed to solve these problems.

ISM formulations basically deal with the emulsification of the internal polymer solution containing the drug with a continuous aqueous or oily phase. The ISMs are formed when the formulation comes in contact with body fluids due to the consequent solidification of the internal polymer. Initial burst release and viscosity were significantly reduced by ISM systems (which is primarily controlled by the external phase). Compared to the use of polymer solutions, painless injectability and abridged pain have been achieved with such systems. In addition, ISMs are multiparticulate which diminish implant morphology dissimilarities with better consistency and reproducibility in the drug release profile (Algın Yapar et al., 2012).

NSAIDs are the corner stone in the management of various chronic inflammatory conditions as rheumatoid arthritis (RA). Through hindering cyclo-oxygenase (COX) enzymes that are responsible for inflammation and synthesis of prostaglandins which are part of the in normal physiological operations. The major adverse effects experienced with the clinical use of NSAIDs are mainly the overdose toxicity. It is believed that the more advanced COX-2-selective moieties better inhibit the inducible form of COX rather than the enzyme's other forms (Bertolini et al., 2002). Oral administration of these agents is the most common, but systemic side effects may be associated with them. Localized application of these compounds is drawing ultimate attention to solve these side effects (Izar et al., 2016).

The aim of the current work was the development, characterization and optimization of targeted, *in situ* forming nano particles (ISNs) for the IA delivery of piroxicam as a model NSAID utilizing full factorial experimental design using Design Expert^®^ 11 (Stat-Ease, USA). Furthermore, the biological performance of the prepared formulations was studied in arthritic rats where histopathological and biochemical studies were performed to investigate efficacy of the proposed delivery systems.

## 2. Materials and methods

### 2.1. Materials

Piroxicam (PX) was a kind gift from Medical Union Pharmaceutical (MUP) Co., Egypt. Tween^®^ 80 (polyethylene sorbitan monooleate), Span^®^ 80 (Sorbitan monooleate), Brij 52^®^ (Polyethylene glycol hexadecyl ether, Polyoxyethylene (2) cetyl ether) and cellulose membrane dialysis bag were purchased from Sigma-Aldrich Chemie GmbH, Steinheim, Germany. Freund's complete adjuvant (CFA) was obtained from Difco Laboratories, Detroit, MI, USA. Captex^®^ GTO was a kind donation from Abitec Corporation, Janesville, USA. Di-methyl sulfoxide (DMSO) was obtained from Merck KGaA, Darmstadt, Germany. Di-sodium hydrogen orthophosphate anhydrous (Minimum Assay Acidimetric 98%), sodium di-hydrogen orthophosphate-1-hydrate (Minimum Assay 98%) and sodium chloride were obtained from ADWIC, Egypt. PURASORB^®^ PDLG 7502 (75/25 DL-lactide/glycolide copolymer) was a kind gift From Corbion Co., Netherlands. Gelatin was purchased from Alnasr Co., Egypt. Eudragit^®^ RL 100 (N,N-dimethylmethanamine;2-methylprop-2-enoic acid) was obtained from Evonik Operations GmbH, Germany

### 2.2. Methods

#### 2.2.1. Preparation of PX-loaded ISNs

PX-loaded ISNs were prepared using the emulsification method(Ammar et al., 2018). The internal phase was prepared by dissolving accurate amounts of PDLG, gelatin, Brij 52 and Eudragit RL in DMSO at 65 °C ± 0.5 °C in an incubation shaker stirrer (IKA Ks4000ic, Germany) at a rate of 180 stroke/minute overnight. The internal phase was stabilized by addition of a precise amount of tween 80. Accurately weighed amount of PX (20 mg) was incorporated into the internal phase and well vortexed till efficiently dispersed (solubility of drug in DMSO ≈ 20 mg/mL) (Castro et al., 2018). The external phase was prepared by mixing exact amounts of Captex^®^ GTO and Span^®^ 80 using vortex mixer (Velp Scientifica, Italy). Finally, the external phase was well vortexed into the internal phase until the emulsion was obtained.

##### 2.2.2. 3^3^ Full Factorial experimental design

PX-loaded ISNs were prepared using a 3^3^ full factorial experimental design utilizing Design Expert^®^ 11 software (Stat-Ease, USA) to explore the combined impact of independent formulation variables. Three inputs were assessed as independent variables at three levels each. The independent variables were: (A) percentage of internal phase, (B) percentage of gelatin and (C) percentage of PDLG. The dependent factors were particle size (PS), polydispersity index (PDI), mean dissolution time (MDT), the release rate constant (K), half-life (T_50%_) and the time needed for quarter of the drug concentration to be released (T_25%_). Experimental trials were performed at all the 27 possible combinations (Table 1).

**Table 1.** Dependent and independent variables of the 3^3^ full factorial experimental design of the piroxicam loaded in situ forming nanoparticles (ISNs).

Formulae	Independent factors			Dependent factors					
	Internal phase %*	Gelatin %	PDLG %	Particle size Mean ± SD (nm)	PDI Mean ± SD	K Mean ± SD (mg.hr^−1/2^)	T_50%_ ± SD (h)	T_25%_ ± SD (h)	MDT ± SD (h)
ISN-1	10	2.5	2.5	283.45 ± 4.172	0.825 ± 0.078	27.67 ± 0.21	2.62 ± 0.11	0.056 ± 0.012	5.26 ± 0.20
ISN-2	10	2.5	5	875.9 ± 105.6	0.95 ± 0.071	23.77 ± 0.43	2.90 ± 0.14	0.038 ± 0.010	5.59 ± 0.51
ISN-3	10	2.5	10	313.3 ± 17.82	0.711 ± 0.127	32.19 ± 0.17	2.21 ± 0.02	0.006 ± 0.001	3.95 ± 0.03
ISN-4	10	5	2.5	178.15 ± 9.687	0.34 ± 0.062	27.47 ± 0.34	2.54 ± 0.10	0.179 ± 0.062	3.36 ± 0.13
ISN-5	10	5	5	232.95 ± 9.829	0.346 ± 0.005	25.65 ± 0.23	2.74 ± 0.19	0.007 ± 0.002	8.16 ± 0.62
ISN-6	10	5	10	194.7 ± 4.243	0.606 ± 0.225	30.19 ± 0.11	2.36 ± 0.03	0.005 ± 0.001	6.42 ± 0.17
ISN-7	10	10	2.5	317 ± 75.24	0.36 ± 0.003	25.28 ± 0.32	2.36 ± 0.12	0.103 ± 0.021	6.65 ± 0.78
ISN-8	10	10	5	230.2 ± 2.121	0.672 ± 0.028	28.84 ± 0.03	2.48 ± 0.06	0.012 ± 0.004	3.25 ± 0.23
ISN-9	10	10	10	258.4 ± 30.26	0.559 ± 0.069	20.81 ± 0.30	2.87 ± 0.23	0.017 ± 0.003	6.69 ± 0.11
ISN-10	20	2.5	2.5	270.25 ± 6.576	0.41 ± 0.09	27.80 ± 0.26	2.41 ± 0.08	0.166 ± 0.055	1.02 ± 0.01
ISN-11	20	2.5	5	552.15 ± 248.5	0.863 ± 0.096	31.99 ± 0.15	2.21 ± 0.24	0.041 ± 0.003	2.15 ± 0.02
ISN-12	20	2.5	10	403.15 ± 13.93	0.542 ± 0.057	29.28 ± 0.06	2.42 ± 0.01	0.028 ± 0.002	0.50 ± 0.08
ISN-13	20	5	2.5	244.3 ± 6.364	0.409 ± 0.057	31.77 ± 0.22	2.28 ± 0.22	0.113 ± 0.032	8.37 ± 0.91
ISN-14	20	5	5	146.5 ± 4.95	1 ± 0	32.81 ± 0.40	2.20 ± 0.16	0.057 ± 0.019	1.93 ± 0.03
ISN-15	20	5	10	191.9 ± 20.79	0.605 ± 0.147	37.43 ± 0.11	2.10 ± 0.03	0.022 ± 0.002	12.72 ± 0.98
ISN-16	20	10	2.5	329.25 ± 30.9	0.383 ± 0.076	31.32 ± 0.29	2.65 ± 0.07	0.033 ± 0.005	5.28 ± 0.34
ISN-17	20	10	5	188.7 ± 49.64	0.439 ± 0	27.32 ± 0.33	2.85 ± 0.17	0.012 ± 0.001	3.91 ± 0.12
ISN-18	20	10	10	235.05 ± 15.63	0.372 ± 0.055	30.77 ± 0.28	2.69 ± 0.20	0.085 ± 0.003	4.12 ± 0.06
ISN-19	30	2.5	2.5	368.25 ± 112.6	0.497 ± 0.063	42.35 ± 0.17	2.11 ± 0.13	0.130 ± 0.021	5.44 ± 0.37
ISN-20	30	2.5	5	470.05 ± 42.36	0.908 ± 0.104	28.72 ± 0.07	2.41 ± 0.21	0.280 ± 0.030	11.01 ± 0.79
ISN-21	30	2.5	10	209.85 ± 52.11	0.692 ± 0.178	36.65 ± 0.66	2.13 ± 0.02	0.343 ± 0.021	2.38 ± 0.05
ISN-22	30	5	2.5	220.3 ± 30.55	0.796 ± 0.138	39.06 ± 0.38	2.04 ± 0.22	0.098 ± 0.014	3.39 ± 0.13
ISN-23	30	5	5	235.95 ± 46.74	0.71 ± 0.088	40.72 ± 0.19	2.00 ± 0.9	0.404 ± 0.002	6.95 ± 0.08
ISN-24	30	5	10	347.4 ± 42.57	0.304 ± 0.042	31.71 ± 0.36	2.42 ± 0.15	0.443 ± 0.023	8.76 ± 0.57
ISN-25	30	10	2.5	350.4 ± 58.83	0.533 ± 0.17	23.10 ± 0.25	3.52 ± 0.06	0.175 ± 0.018	15.38 ± 1.2
ISN-26	30	10	5	364.9 ± 42.57	0.385 ± 0.028	20.96 ± 0.13	3.66 ± 0.05	0.250 ± 0.007	46.12 ± .07
ISN-27	30	10	10	1310 ± 130.1	0.635 ± 0.073	20.69 ± 0.67	3.70 ± 0.26	0.624 ± 0.033	17.55 ± 0.98

PDLG: PURASORB® PDLG 7502- 75/50 DL-lactide/glycolide copolymer; PDI: poly dispersity index; K: release rate constant; T_50%_ and T_25%_: time required for 50 and 25% of the drug to be released, respectively; MDT: mean dissolution time.

*Internal phase contains 2 mg of piroxicam in all formulations stabilized by 0.1% tween 80.

#### 2.2.3. Characterization of the prepared PX-ISNs

##### 2.2.3.1. Particle size and PDI (polydispersity index) determination

To determine both the particle size and the PDI of the ISNs, 1 ml of the formula was diluted in a 1:10 ratio using deionized water and stirred on heated magnetic stirrer (Velp-AREC.T F20500051, Velp Scientifica, Italy) for 1 hr. In a cooling ultracentrifuge (3-30KS, Sigma laborzentrifugen, Germany), the prepared sample was centrifuged (15000 rpm, 4 °C) for 15 minutes followed by the removal of oily phase. The particles were suspended in 1 mL deionized water and particle size was measured. Dynamic light scattering (DLS) analysis using ZetaSizer (Nano Zs, Malvern instruments limited, UK) (*n* = 3) SD was used to determine the average particle size and size distribution of the obtained vesicles.

##### 2.2.3.2. *In-vitro* drug release study

Dialysis bag method was utilized for the study *in-vitro* release profile of PX from the prepared formulae, with a cellulose membrane dialysis bag (dimensions 7 cm length, 2.2 cm wide and molecular weight cut off 12–14,000 Daltons) serving as the donor compartment (Aggarwal & Kaur, 2005). Accurately measured 0.5 ml PX-loaded ISNs were injected into the dialysis bag then fitted in 100 mL phosphate-buffered saline (pH 7.4) resembling the receiving compartment and incubated at a fixed temperature of 37 ± 0.5 °C in an incubation shaker at a rate of 180 rpm. To maintain the sink conditions, 5 mL of the release medium were taken and substituted by fresh medium at predetermined time intervals(Jamzad & Fassihi, 2006; Phillips et al., 2012). The drug content of the withdrawn samples was measured spectrophotometrically at a predetermined wavelength. Average cumulative PX released percentage was charted versus time and the release data were kinetically investigated by fitting the data into different kinetic models namely; zero and Higuchi diffusion release models, using linear regression analysis in order to find the best fit of the release data followed by confirmation of the obtained results using Korsmeyer-Peppas equation to determine the mechanism of drug release from the prepared formulations (Chandra Basak et al., 2008; Onnainty & Granero, 2019). Different release parameters were calculated to compare the investigated formulations. The investigated release parameters involved the time needed for quarter of the drug concentration to be released (T_25%_), half-life (T_50%_), the release rate constant (K) and the mean dissolution time (MDT).

##### 2.2.3.3. Selecting the optimized formulations

The desirability function of the Design Expert^®^ software was utilized to select the optimized formulations for further investigations. The targeted criteria were to maximize MDT, T_25%_ and T_50%_ and to minimize PS. Only the significant models were included except for the particle size that was included although being insignificant due to its major impact on the retention period as well as the decreased clearance at the site of injection.

##### 2.2.3.4. Characterization of the optimized formulation

###### 2.2.3.4.1. Transmission electron microscopy (TEM)

Optimized ISNs were subjected to morphological examination using TEM (High-resolution Transmission electron microscope) (HR-TEM) - JEOL2100-USA, Wilmington, DE, USA). The obtained emulsions were injected into 10 mL phosphate buffer (pH 7.4) and kept in an incubation shaker for 24 hrs to ensure the formation of nanoparticles followed by centrifugation (15000 rpm, 4 °C) for 15 minutes, afterward the oily phase was removed(Ammar et al., 2018). The nanoparticles were dispersed in 1 mL phosphate-buffered saline (pH 7.4) then morphology was examined using a Transmission electron microscope with an accelerating voltage of 80 kV. A drop of the produced particles was positioned onto a carbon-coated copper grid and allowed to adhere for roughly 2 minutes. A drop of phosphotungstic acid solution (2% *w/v*) was layered onto the carbon grid. Finally, the sample was air dried before the thin film of dyed ISNs was examined. (Eltellawy et al., 2021).

###### 2.2.3.4.2. Fourier Transform Infrared Radiation Measurements (FT-IR)

FT-IR analysis was performed on pure PX powder, gelatin, PDLG, Brij 52, Eudragit RL 100 and their physical mixture. Additionally, FT-IR analysis was conducted on the optimized drug-loaded and unloaded formulations. The samples were analyzed using an FT-IR spectrophotometer (Model 22, Bruker, UK) using the KBr disk technique, which was performed at ambient temperature in the scanning range of 4000–400 cm^−1^. A mercury cadmium telluride detector was used to collect the spectra, which had a resolution of 4 cm^−1^. The samples were tested three times (Titus et al., 2019).

###### 2.2.3.4.3. Viscosity measurements

The viscosity of the selected ISNs was determined at 25 °C ± 0.2 °C, using a computer-linked up Brookfield rheometer (DV3THB cone/plate rheometer, spindle CPE-40, and RheocalcT software, version 1.1.13 software) (PolyScience model 9006, USA). A plate and cone configuration with a 20 mm diameter/4° angle and a fixed shear rate (1/s) was used to measure the viscosity of the ISNs preparation. The rheological performance of the formulations was calculated by charting the shear stress against the shear rate values. According to Farrow's equation, flow behavior was investigated:

Log  D=N  Log  S− Log  η



where, D represents the shear rate (sec^−1^) and S stands for the shear stress (Pa). N is Farrow’s constant while η represents the viscosity (Pa.s). N stands for the slope of the charting of log D versus log S, which designates the deviance from Newtonian flow. When N has a value of less than unity, it ensures dilatant flow which is a shear-thickening flow, while values greater than unity specify pseudoplastic or plastic flow which are shear-thinning flows.

###### 2.2.3.4.4. Injectability

In an attempt to assess the formulation's ease of injection, injectability testing was done on the optimized formulation comparing its performance to a commercialized oily injection, Betolvex TM, using a home-modified device (Shamma et al., 2017). The designed apparatus was similar to what had been described previously by Jones and Leroux, 1999 but with a few modifications (Jones and Leroux, 1999). One mL of the evaluated formulation was placed in a syringe of 5 mL volume fitted with a 19 gauge needle. The filled syringe was then fixed to a rubbery tube that ended in an air pump. Injectability was measured by forcing air onto the solution surface from an air pump. Using a sphygmomanometer, the pressure applied on the surface of the solution was measured in mmHg units and retained steady at 70 mm Hg (Shamma et al., 2017). The time needed for 1 mL of the sample to be released was noted. The flow-rate values (mL/min) were contrasted to conclude the injectability of the investigated sample (Yehia et al., 2012).

###### 2.2.3.4.5. *In-vivo* evaluation of selected PX-ISNs

####### 2.2.3.4.5.1. Experimental design

Thirty-six adult male Sprague–Dawley rats (mean body weight of 200 ± 50 g) were utilized in the experiment. The rats were reserved under well-defined and standardized conditions (humidity- and temperature-controlled room; 12-h light and 12-h dark cycle). They were fed a typical rat regimen with unlimited water supply. Animals were set to a two-week adaptation period preceding the experiments. Ethical Guidelines for Laboratory Animal Research were strictly followed, and the experiments were permitted by the Ethical Committee of the Faculty of Pharmacy, Future University in Egypt, Egypt (REC-FPSPI-14/107) and in accordance with use of Laboratory Animals of the National Institutes of Health (NIH Publications No. 80-23, revised 2011). Unnecessary animal disturbances had been prevented. Gentle management of the animals was achieved during treatment through avoiding pain, squeezing, and any tough handling. In order to prompt arthritis in rats, 0.1 mL Complete Freund’s adjuvant (CFA; 1 mg/ml) was subcutaneously injected into the left hind paw plantar surface. An intra-dermal booster dose of 0.1 ml was injected through the tail root on the day of the experiment as well as the following day (Kamel et al., 2018).

The rats were randomly distributed into six groups (*n* = 6) as follows: Group A: received no injection (Normal control). Group B: received CFA as described under induction of arthritis section and served as arthritic disease control (Kamel et al., 2018). Group C: arthritic diseased rats and after 2 weeks they were treated with oral drug solution based on a once-daily regimen for 30 days. Group D: arthritic diseased rats treated with intra-articular drug solution (0.1 ml) once weekly for four successive weeks. Group E: arthritic diseased rats treated with optimized formulation (0.1 ml) once weekly for four successive weeks. Group F: arthritic diseased rats treated with placebo formulation (0.1 ml) once weekly for four successive weeks. Piroxicam dose (10 mg/kg) was used based on previously published data (Uttra et al., 2018; Saleem et al., 2020).

####### 2.2.3.4.5.2. Preparation of samples

Approaching the termination of the treatment period, blood samples were obtained from the retroorbital plexus under anesthesia and sera were separated for the determination of anti-cyclic citrullinated peptide (anti-CCP) and monocyte chemoattractant protein-1 (MCP-1) levels. Animals were then euthanized by an overdose injection of thiopental. The left hind limbs were rapidly isolated and weighed. Two limbs from each group were preserved in neutral formalin (10% *v/v*) to be assessed later for histolopathological investigation to determine the impact of various treatments on RA-induced histopathological changes. Furthermore, limbs from groups showing most improvement were further homogenized to complete the mechanistic study. The contents of matrix metalloproteinase-3(MMP-3), interleukin-17 (IL-17), nuclear factor κ-B (NF-κB) and tumor necrosis factor-α (TNF-α) were determined. In addition to the evaluation of the relative protein expression of receptor activator of nuclear factor kappa-Β ligand (RANKL) and signal transducer and activator of transcription 3 (STAT-3).

####### 2.2.3.4.5.3. Histopathological examination

Left hind limbs (knees and paws) were kept in 10% neutral formalin to be fixed for 48 h., followed by addition of 4 M formic acid for decalcification for 35 days. Tap water was used for washing, followed by addition of a serially diluted blend of methyl, ethyl and absolute ethyl alcohols to obtain dehydration. Xylene was utilized for sample clearing in and was subsequently embedded in paraffin kept at a temperature of 56 °C in a hot air oven for a duration of 24 h. A combination of paraffin and bees wax was used to prepare the tissue blocks to be sectioned at a thickness of 4 μm using sledge microtome. Collection of the tissue samples was done on glass slides, then deparaffinization was pursued, and the staining was done by means of eosin and hematoxylin. A Light electric microscope was employed for the examination of the samples (Suvarna et al., 2013).

####### 2.2.3.4.5.4. Assessment of the protein expression of STAT-3 and RANKL

For determination of the protein expression of STAT-3 and RANKL, the western blot method was employed (Mansour et al., 2021). Phosphate buffered saline was utilized for the homogenization of the obtained hind limb tissues. Followed by using SDS polyacrylamide gel electrophoresis for the separation of exactly 10 μg protein from each limb sample that are then moved to a nitrocellulose membrane. The obtained membrane was incubated with either anti-RANKL (cat# MBS8813030) or anti-STAT-3 (cat# MBS8808638) antibodies (MybioSource, CA, USA) for 24 hours at 4 °C and the formed blots were examined using enhanced chemiluminescence detection reagent (Amersham Biosciences, IL, USA). The obtained outputs were conveyed as arbitrary units against β-actin (cat# MBS448085) employing image analysis software (Image J, version 1.46a, NIH, Bethesda, MD, USA).

####### 2.2.3.4.5.5. Determination of serum level of anti-CCP and MCP-1, as well as hind limb contents of IL-17, MMP-3, NF-κB and TNF-α

Serum levels of anti-CCP (MBS226116) and MCP-1 (MBS8804486), as well as IL-17 (MBS8801559), TNF-α, (MBS2507393), MMP-3 (MBS8801537) and NF-κB (MBS8804668) hind limb contents were measured using their corresponding ELISA kits (MybioSource, CA, USA) following the manufacturer’s instructions.

##### 2.2.3.5. Statistical analysis of data

The obtained data were presented as mean ± SD (standard deviation). For the analysis of the full factorial experimental design to investigate the effect of independent variables on the dependent ones, Design-Expert^®^ 11 software (Stat-Ease, Inc., USA) was used, with subsequent ANOVA testing to investigate the statistical significance and the impact of the independent variables on the dependent ones. In all experiments, the statistical level of significance was set at *p* <.05. One-way ANOVA followed by Tukey multiple comparison tests was performed using GraphPad Prism 8.0^®^ software (GraphPad Software, San-Diego, CA, USA) for all other statistical analyses.

## 3. Results and discussion

### 3.1. *In-vitro* drug release studies

One of the most important studies for all controlled release delivery systems is the release study. It is an eminent way to filter out systems that do not have the desired release profiles. *In-vitro* tests are extremely useful for checking the quality of the finished systems (Guyot & Fawaz, 2000).

**Figure 1.**
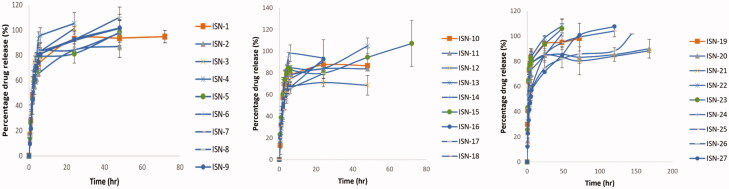
Release profile of the prepared ISNs.

All formulations exhibited a dual phased release pattern, with an initially fast release phase followed by a more delayed-release phase that extended up to 144 hours in some formulations as shown in Figure 1. Upon contact of the formulation with the release medium, the internal phase solvent diffuses through the external oily phase leading to the deposition of the nanoparticles and the entrapment of the drug. The biphasic release is a consequence of the presence of some unentrapped drug that was not incorporated into the core of the formed particles which exhibits a faster release compared to the entrapped drug (Ammar et al., 2020).

The release parameters studied were mean dissolution time (MDT), the release rate constant (K), half-life (T_50%_) and the time needed for quarter of the drug concentration to be released (T_25%_).

#### 3.1.1. Assessment of *in-vitro* drug release kinetics

Average cumulative drug released percent was charted versus time as shown in Figure 1, and the release data were kinetically analyzed by substituting the obtained release data into different kinetic models, such as the zero and Higuchi diffusion release models, using linear regression analysis to find the best fit of the release data, and then confirming the results using the Korsmeyer-Peppas equation to detect the mechanism of drug release from the investigated formulae (Thapa et al., 1970; Chandra Basak et al., 2008; Onnainty & Granero, 2019). Different release parameters were computed to assess the differences between the prepared formulations. The Higuchi diffusion release model fitted into all formulations’ data. All analyzed formulations were found to follow non-fickian transport except for ISN-19, where the value of n was less than 0.5, indicating fickian transport. A combination of a diffusion and chain relaxation mechanism was found to control non-fickian release. The chain relaxation mechanism can be likened to the structure of gelatin, which contains a long carbon chain, through the relaxation of polymer sections between network nodes (Bacaita et al., 2014). The diffusion process, on the other hand, could be attributed to polymer degradation and surface erosion due to the existence of PDLG (Jusu et al., 2020).

### 3.2. Statistical analysis of the 3^3^ full factorial experimental design

#### 3.2.1. Effect of formulation factors on the T_25%_

**Figure 2.**
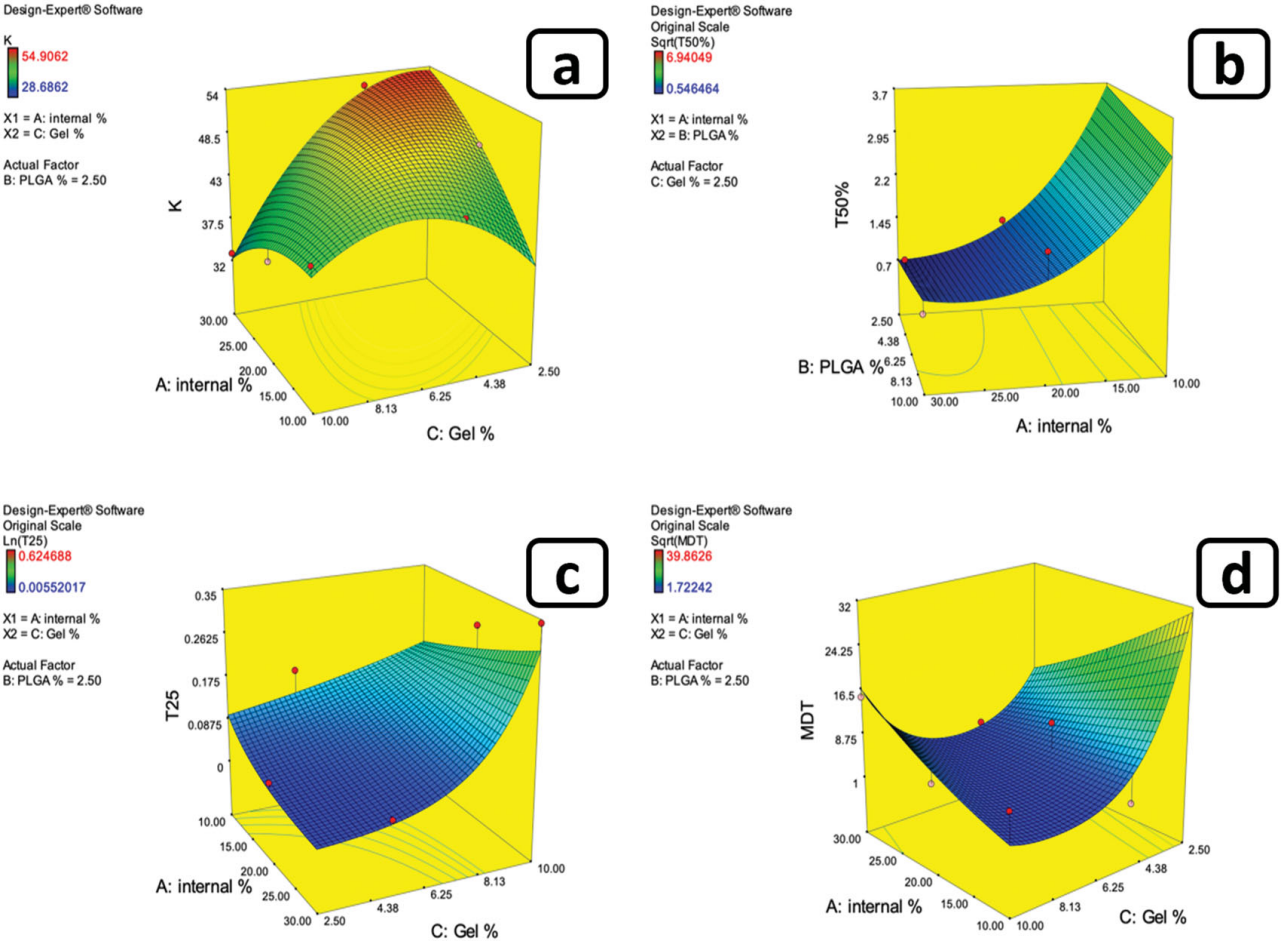
Example of 3D response surface plots for the effect of formulation factors on (a) K, (b) T50%, (c) T25%, and (d) MDT.

**Figure 3.**
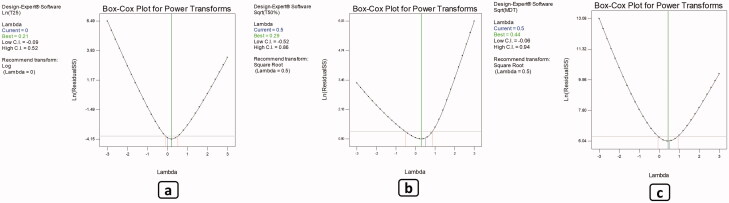
Box–Cox transformation for (a) T25%, (b) T50%, and (c) MDT.

ANOVA test was executed to assess the degree of significance of the investigated independent factors on T_25%_ of the drug which indicates the length of time required for quarter of the amount of the drug to be released. Table 1 shows the values of the measured response and Table 2 presents the model regression analysis. The adopted model exhibited good correlation between the values of the *R^2^* (0.8781), and adjusted *R^2^* (0.8259) and predicted *R^2^* (0.7158), as well as the adequate precision of value 13.055, which ensures the model adequacy and adequate signal which assures that the current model can be utilized to explore the whole design space. Box-Cox transformation was employed as a diagnostic test to assure if there was a necessity for power transformation of the model to better interpret the results and improve the skewed variables while creating a model equation that best fits the data(Box & Cox, 1964; Osborne, 2010). The selected model needed further transformation, where the current lambda was superimposed with the best lambada (lambda = 0.0) as shown in Figure 3. Results showed that only gelatin percentage (C) significantly (*p* < *.0001*) influenced the obtained values of (T_25%_) of the drug. Also, the two-factor combinations AB and AC had a significant (*p =* *.0018* and *p* = *.0032*, respectively) effect on (T_25%_) values (Figure 2). The change of the percentage of gelatin from the lower level to the higher level had significantly (*p* < *.0001*) increased the (T_25%_) values which is a reflection of the delay of the drug release and the control of the well-known burst effect accompanied with this type of formulations. Furthermore, the upsurge in the inner phase volume is accompanied with the consequent augmentation in the total amount of gelatin available. This in turn will favorably retard the diffusion of the internal phase solvent which has a good miscibility with water and consequently the deposition of the particles. Additionally, the presence of Brij 52 with its large polar head which increases the polarity of the internal phase retarding the diffusion of DMSO through the external phase and the deposition of the ISNs. This is similar to the findings of Ammar et. al. who reported the effect of the polar head of Brij 52 on the retardation of the internal phase diffusion and the deposition of the *in-situ* forming vesicles (Ammar et al., 2018). All of which gives more room for the incorporation of both the drug and gelatin into the ISNs. Moreover, the increase in the percentage of PDLG, being he integral particle producer, accompanied with the increase in internal phase %, added to the formation of more particles, thus incorporation of both gelatin and drug into the core of the particles decreasing the amount of the free drug. Keeping in consideration that the remaining unentrapped gelatin will still serve to increase the viscosity of the surrounding medium upon contact with the adjacent aqueous medium. This is maybe due to the fact that gelatin has the ability to swell and absorb water upon contact with dissolution medium and forming a gel matrix, so increasing the concentration of gelatin increases the viscosity of the formed matrix resulting in slow drug diffusion through the gel matrix (Okino et al., 2002). As a result, when ISNs become embedded in a gelatin-based solution after deposition, their stability and viscosity will improve, as did the loaded drug's half-life. Within the gaps between the polymer crosslinks, gelatin hydrogels can trap the free drug that failed to load inside the deposited ISNs. This is in accordance with the results reported by Foox et. al. who observed the prolongation of the drug half-life following its formulation into gelatin hydrogel(Foox & Zilberman, 2015).

**Table 2.** Model parameters of the 3^3^ full factorial experimental design of the piroxicam loaded in situ forming nanoparticles (ISNs).

Model parameters	T_25%_ (h)	T_50%_ (h)	K (mg.h^−1/2^)	MDT (h)
Model type	2FI model	Quadratic model	Quadratic model	Quadratic model
*R* ^2^	0.8781	0.9076	0.8868	0.7047
Adjusted *R^2^*	0.8259	0.8459	0.7942	0.5781
Predicted *R^2^*	0.7158	0.6757	0.6166	0.3045
Adequate precision	13.055	13.663	10.488	7.833
Final Equation in terms of coded factors	Ln(T25) =	Sqrt (T50%) = +0.95	K = +48.73	Sqrt(MDT )=
	–2.64	+0.065 * A	–0.20 * A	+2.87
	+5.183E–003 * A	+0.042 * B	–0.58 * B	+0.071 * A
	–0.052 * B	+0.28 * C	–5.20 * C	+0.48 * B
	+1.44 * C	+0.095 * A * B	–2.21 * A * B	–0.82 * C
	+0.59 * A * B	+0.51 * A * C	–6.17 * A * C	+1.13 * A * C
	+0.81 * A * C	+0.019 * B * C	–0.77 * B * C	–0.60 * B^2
	+0.30 * B * C	+0.26 * A^2	–2.25 * A^2	+1.92* C^2
		+0.47 * C^2	–0.018 * B^2	
			–6.14 * C^2	

K: release rate constant; T_50%_ and T_25%_: time required for 50 and 25% of the drug to be released, respectively; MDT: mean dissolution time; *R^2^*: squared regression coefficient.

#### 3.2.2. Effect of formulation factors on the half-life of the drug (T_50%_)

The implication level of the investigated factors on the drug's half-life was determined using the ANOVA test. The half-life indicates the length of time required for the release of half of the initial drug concentration (Tables 1 and 2). The adopted model exhibited good correlation between the values of the *R^2^* (0.9076), and adjusted *R^2^* (0.8459) and predicted *R^2^* (0.6757), as well as the adequate precision of value 13.663, which ensures the model adequacy and adequate signal which assures that the current model can be utilized for the navigation of the whole design space. Box-Cox transformation was employed as a diagnostic test to assure if there was a necessity for power transformation of the model to better interpret the results and improve the skewed variables while creating a model equation that best fits the data (Box & Cox, 1964; Osborne, 2010). The selected model needed further transformation, where the current lambda was superimposed with the best lambada (lambda = 0.5) as shown in Figure 3.

Results showed that only gelatin percentage (C) significantly (*p = .0004*) influenced the half-life of the drug (T_50%_). Also, the two-factor combination AC had a substantial (*p = <.0001*) impact on (T_50%_) values (Figure 2). As previously explained with T_25%_ results, both the presence of gelatin and its effect as a viscosity enhancer and the presence of the large polar-headed Brij 52 benefited in augmenting the incorporation of the drug into the deposited ISNs. Added to the increased amount of PDLG in the internal phase which supported the formation of particles. Additionally, the FTIR findings suggested the formation of a hydrogen bonding between PX and both the gelatin and Brij 52 which consequently retarded the release of the drug from the core of the formed particles reflected in a significant increase in T_50%_.

#### 3.2.3. Effect of formulation factors on the mean dissolution time (MDT)

Using ANOVA test, the significance level of the investigated factors on the MDT values was evaluated as shown in Tables 1 and 2. Box-Cox transformation was employed as a diagnostic test to assure if there was a necessity for power transformation of the model to better interpret the results and improve the skewed variables, while creating a model equation that best fits the data (Box & Cox, 1964; Osborne, 2010). The selected model needed further transformation, where the current lambda was superimposed with the best lambada (lambda = 0.5) as shown in Figure 3. Results showed that the percentage of gelatin (C) had significant (*p = .0065)* effects on the MDT values. Additionally, the two-factor combination AC significantly (*p = .0044)* affected the MDT values (Figure 2). As a consequence to the previously explained significant prolongation of both T_25%_ and T_50%_ that was credited to the increase in gelatin concentration as well as the increase in PDLG, Brij 52 and internal phase percentage, the rate of ISNs deposition was reasonably augmented. This consequently increased the entrapment of both the gelatin and the drug into the particles which in turn minimized the initial release (reflected in the significant decrease in T_25%_). Furthermore, the unentrapped gelatin controlled the initial release of the drug and down surged the burst effect. In addition, the probability of hydrogen bond formation between the drug and the particle components added to the prolongation of drug release. All of which sums up into the overall retardation of drug release and the significant (*p = .0039*) increase in MDT values.

#### 3.2.4. Effect of formulation factors on the release rate constant (K)

The effect of the tested factors on the release rate constant which indicates the rate of diffusion of the drug through the nanoparticles matrix was evaluated through the ANOVA test as shown in Tables 1 and 2. The generated model exhibited good correlation between the values of *R^2^* (0.8868), and adjusted *R^2^* (0.7942) and predicted *R^2^* (0.6166), as well as the adequate precision of value 10.488, which ensures the model adequacy and adequate signal which assures that the efficacy of the generated model to investigated the entire design space. The selected model needed no further transformation, where the current lambda was effective with no need for further data transformation, where the current lambda (1.00) laid within the 95% confidence interval of the best lambda value. Results showed that only gelatin percentage (C) significantly (*p = .0003*) affected the release rate constant (K). Also, the two-factor combination AC significantly (*p = .0009*) influenced (K) values as shown in Figure 2. Results revealed that by increasing the internal phase percentage while decreasing gelatin percentage lead to rapid diffusion of the water miscible internal phase solvent through the external phase, leading to the rapid deposition of the ISNs. This could have resulted in less drug entrapment in the particles, resulting in a higher rate of free drug release.

#### 3.2.5. Effect of formulation factors on average PS and PDI values of the prepared ISNs

On the basis of the ANOVA test results, the effect of formulation factors on the particle size and PDI was insignificant (*p > .05*).

### 3.3. Characterization of the optimized formulation

Based on the desirability criteria adopted and applied using the Design^®^ Expert desirability function, ISN-26 was chosen as the optimized formulation for further investigations.

#### 3.3.1. Transmission electron microscopy (TEM)

**Figure 4.**
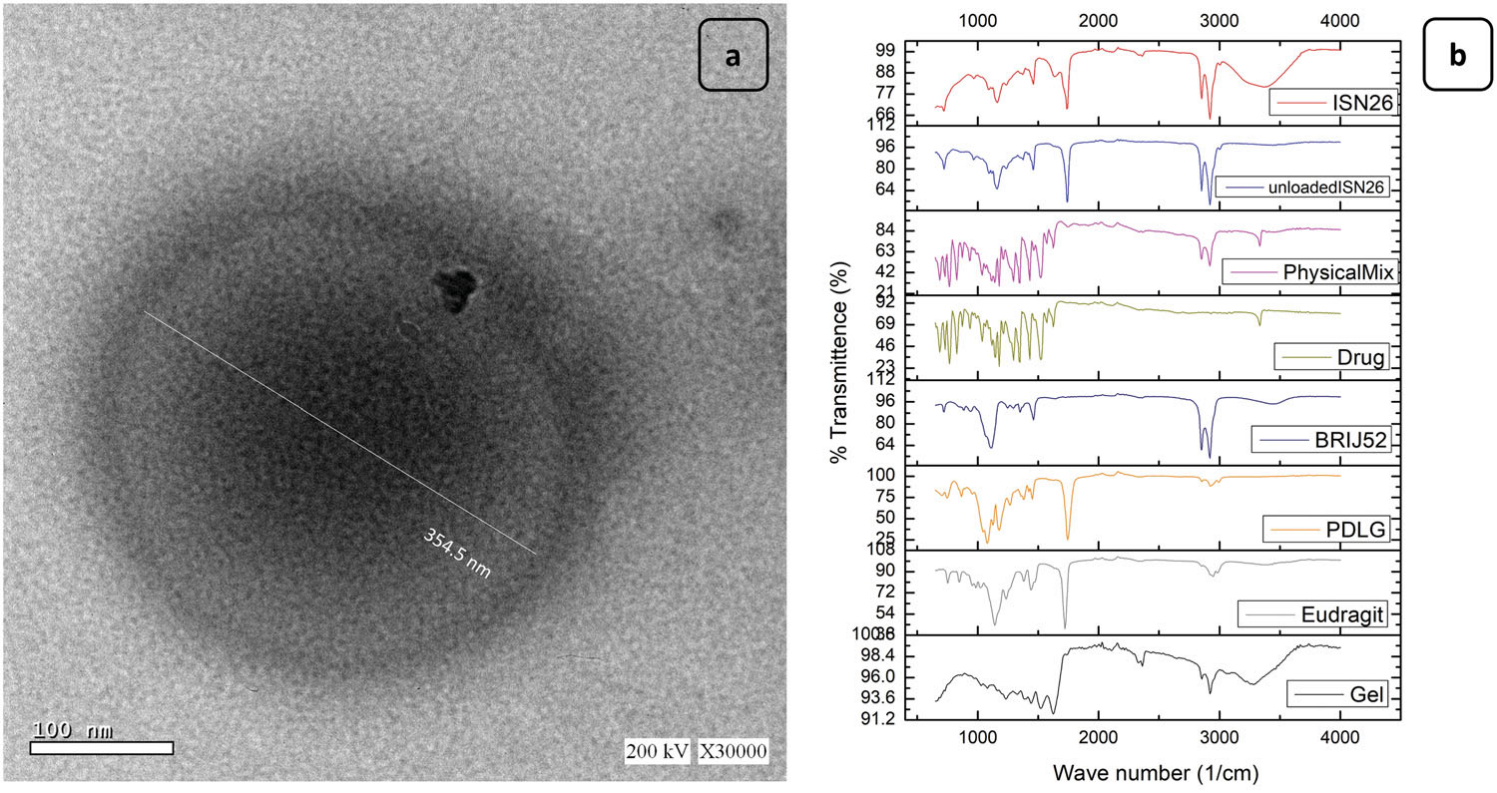
(a) Transmission electron micrograph (TEM) of the optimized formulations (ISN-26). (b) Fourier transform infrared radiation (FT-IR) spectrums of ( from bottom to top) pure Gelatin, Eudragit RL 100, PDLG, Brij 52, PX powder, their physical mixture, the unloaded ISN-26 and the PX-loaded ISN-26.

TEM was utilized to determine the morphological pattern of the optimal formulation. The obtained images revealed the formation of spherical nanoparticles with a dense core and a less dense outline as presented in Figure 4. This may be explained by the deposition kinetics where the particles are formed upon the diffusion of the solvent into the aqueous environment and the deposition of the nanoparticles. The presence of the viscosity regulator gelatin as well as the large polar headed Brij 52, may have retarded the diffusion of the DMSO into the release medium resulting in the formation of the dense particles. Additionally, the incorporation of gelatin into the core of the particles may have densified the interior of the obtained particles. The less dense outline, on the other hand, could be due to the release medium's surface penetration into the vesicles during their deposition especially in the presence of peripherally attached gelatin that swells upon contact with the aqueous environment.

#### 3.3.2. Fourier transform infrared radiation measurements (FT-IR)

The interaction of drug with excipients in the selected formulation was studied by FTIR spectroscopy. The FTIR spectra of the pure PX, PDLG, eudragit RL 100, brij 52 and gelatin as well as their physical mixture and both ISN-26 and its drug free analogue have been depicted in Figure 4. Piroxicam showed vibrational frequency at 3330 cm^−1^ which is assigned to pyridin-2-yl-amino stretching. Vibrational peaks at 1628, 1575–1560, and 1520 cm^−1^ were due to amide C=O stretching, C=C stretching, and amide-II (N–H) bending, respectively. The FT-IR data of piroxicam were well supported by the literature data. The FTIR spectra of PDLG shows distinctive peaks at 2855 and 2922 cm^−1^ resulting from C–H, C–H_2_ and C–H_3_ stretching vibration whereas the peaks at 1386, 1453 and 1744 cm^−1^ were attributed to bending vibration in relation to the spectrum connected with C=O groups (Singh et al., 2015). The band at 1326 cm^−1^ in GEL is related mainly to the wagging vibration of proline side chains. The N-H stretching produced the amide A band at 3270–3370 cm^−1^ which is relative to the cross-linking degree. Also the C–H stretching at ∼2922 cm^−1^ produced the amide B band, C=O stretching resulted in the amide I at1625 cm^−1^ while the peak at 1500–1520 cm^−1^ represents the amide II N–H deformation (Stevens, 2008). The FTIR spectra of Brij 52 shows characteristics peaks at 3466 cm^−1^ representing the stretching O–H and two peaks 2842 and 2922 cm^−1^ which represents the aliphatic C–H. Finally, the peak at 1110 cm^−1^ represents C–O–C group. The eudragit RL 100 FTIR spectrum revealed peaks at 2952 cm^−1^ representing aliphatic C–H group and another peak at 1722 cm^−1^ which represents the stretching C=O. Comparable peaks were recognized in the spectrum of the drug-free formulation and the physical mixture. Hence, it can be postulated that the drug had no chemical interaction with excipients of the vesicles. However, the disappearance of the N–H peak of gelatin as well as the O–H peak of brij^®^ 52 and the C=O peak of the drug indicates the formation of hydrogen bonding between the drug and both the gelatin and the brij that may have improved the encapsulation of the drug during the deposition of the ISN. Similarly, these hydrogen bonds may have enhanced the incorporation of gelatin into the ISNs that boosted retardation of the drug release.

#### 3.3.3. Viscosity measurements

Aqueous solution or oil-based solutions, emulsions, and suspensions are all types of pharmaceutical parenteral formulations that can be injected intravenously, intramuscularly, intradermally, intralesional, intraarticularly, or subcutaneously. The injectability of injectable suspensions must be determined. The pressure or force required throughout injection, the symmetry of flow, aspiration attributes, and freedom from clogging are all issues to be considered from a therapeutic standpoint.(Schwendeman et al., 2014). As computed from Farrow’s equation, Farrow’s constant was found to be 1.1048 ± 0.001, which confirmed the formulation pseudoplastic properties. The viscosity of the examined formulation decreased with increasing shear rate, and this may be due to polymers presence (Jones et al., 2003) which is beneficial in parenteral controlled release injectable formulations to ease the injection of the formulation followed by an increase in viscosity at the injection site, which prolongs drug release and creates a temporary depot at the injection site.

#### 3.3.4. Injectability

The injectability of the formulas under investigation was determined by comparing the mean flow rates. Under continuous pressure, according to the results of the investigated formulations, the oily marketed sample (BetolvexTM) took a longer time to cross the 19-gauge needle. The average time for Betolvex TM to flow was 62 seconds, and the flow rate was 0.97 mL/min. While the average flow time for a 1 mL ISN-26 injection was 11 seconds, and the flow rate was 5.45 mL/min. This high flow rate of the optimized formulation indicates its good injectability (Berteau et al., 2015). This may be correlated with its observed pseudoplastic flow behavior which facilitates the injectability of the formulation upon applying sufficient pressure.

#### 3.3.5. *In-vivo* evaluation of selected PX ISNs

##### 3.3.5.1. Effect on the CFA-induced histopathological alterations

**Figure 5.**
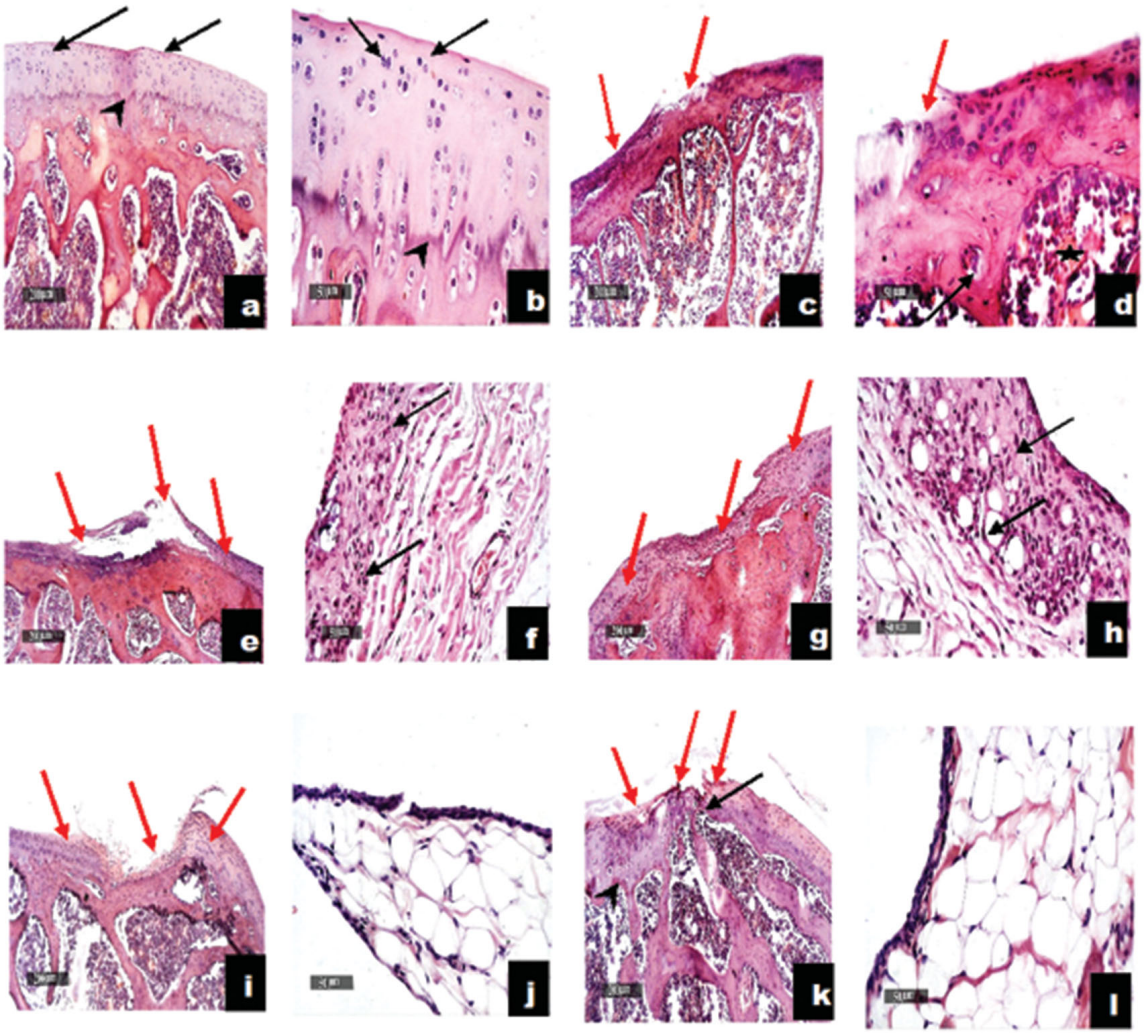
Effect of optimized piroxicam formulation on histopathological alterations of CFA-induced arthritis in rats. Sections c and d of RH rats showing wide areas of articular surface erosions and irregularities with significant loss of chondrocytes at different zones and abundant fibrous tissue replacement rich with inflammatory cells infiltrates (red arrow). Significant decrease of articular cartilage thickness, loss of intercellular matrix staining intensity and demarcation lines with congested subchondral bone marrow blood vessels (star). Moderate to severe mononuclear inflammatory cells infiltrates in synovial membranes (black arrow) as compared to N group (a and b). Sections of oral solution (e and f) and intra-articular suspension (g and h) representing almost same changes with milder inflammatory cells infiltration. Sections of RH + ISN26 Placebo (i and j) group showing focal smaller articular surface erosions and fissures with more organized fibrous tissue and abundant undifferentiated fibroblasts and immature chondroblasts (red arrow). Sections of RH + ISN26 (k and l) group showing more accelerated chondrogenic maturity with abundant mature chondrocytes (black arrow) at lateral borders with significant smaller focal erosion areas (red arrow) and significant restoration of articular cartilage thickness, well demarcated calcified and non-calcified cartilaginous matrix (arrowhead) with significant reduction of inflammatory cells infiltrates in synovial membranes. N: normal, RH: rheumatoid arthritis, ISN26 Placebo: unloaded optimized formulation, ISN26: optimized formulation (100 ×; bar = 200 μm), (400 ×; bar = 50 μm).

As shown in Figure 5, IA administration of CFA resulted in marked joint destruction as manifested by the presence of wide areas of articular erosions and irregularities with marked inflammatory cell infiltration, significant loss of chondrocytes and abundant fibrous tissue replacement. These findings that go in line with many investigators (Kamel et al., 2019; Weng et al., 2021). Similar records were noticed following the administration of piroxicam oral solution and IA suspension with milder inflammatory cells infiltration in the synovial membranes. On the other hand, once a week IA administration of the unloaded optimized formulation alone to CFA-induced RA rats demonstrated some healing properties as evidenced by the abundance of the undifferentiated fibroblasts and immature chondroblasts. Moreover, the optimized piroxicam formulation showed more accelerated chondrogenic maturity with abundant mature chondrocytes at lateral borders with significant smaller focal erosion area with marked restoration of the articular tissue. Beyond to the well-established role of piroxicam in managing RA, the unique amino acid and peptide profile of gelatin may be responsible for observed improvement in RA. This is in agreement with the findings of a research group that reported that orally administered pharmaceutical-grade collagen hydrolysate (PCH) which is derived from the breakdown of gelatin lead to a significant stimulation of synovial cells (Ohara et al., 2010; T et al., 2017). Furthermore, the accelerated chondrogenic maturity upon treatment with the optimized piroxicam formulation may be further attributed to the presence of the positively charged Eudragit polymer and the Gelatin A that acquires positive charge in the physiological pH, both of which may have retarded the clearance of the optimized formulation from the synovial fluid due to the interaction with the negatively charged hyaluronic acid (HA) as a natural component of synovial fluid. This agrees with the findings of Kim et. al. who observed the formation of filamentous aggregates in the micro-meter range attributed to electrostatic interaction between the prepared positively charged PLGA/Eudragit nanoparticles and the HA (Levick, 1998; Edwards et al., 2007; Champion et al., 2008; Kim et al., 2015). Moreover, the particle size of the optimized ISN-26, being in the nano-meter range (364.9 ± 42.57 nm) may have retarded its clearance from the synovial fluid in addition to preventing its phagocytosis. Furthermore, it has been claimed that adminstration of collagen hydrolysates (gelatin) might elaborate the formation of cartilage matrix, by invigorating the chondrocytes (Reginster & Veronese, 2021).

##### 3.3.5.2. Effect of optimized piroxicam formulation on serum levels of anti-CCP and MCP-1

**Figure 6.**
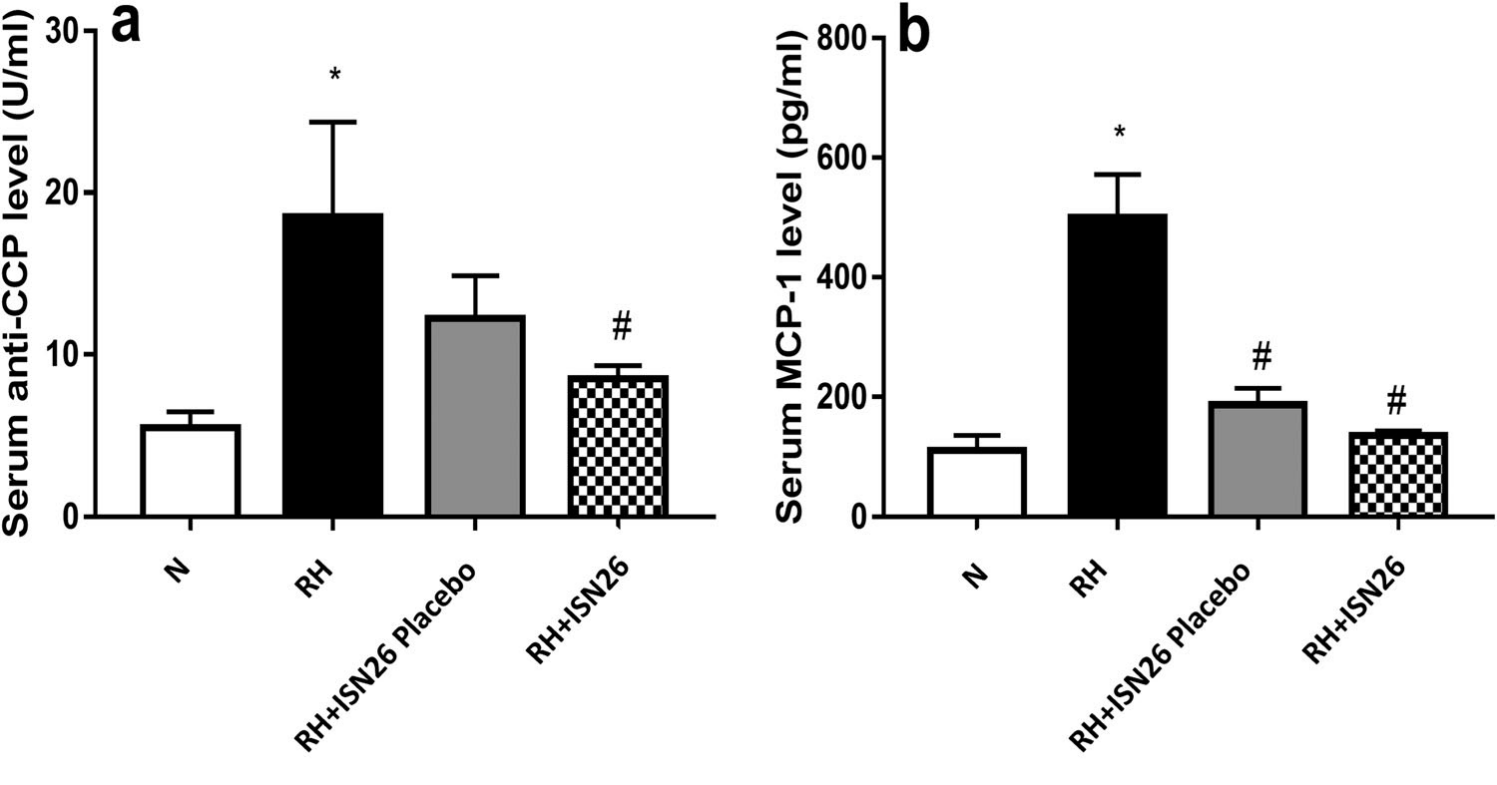
Effect of intra-articular optimized piroxicam formulation on serum levels of a) anti-CCP and b) MCP-1 in CFA-induced arthritis in rats. Data are expressed as mean ± S.D. Statistical analysis was done using one-way ANOVA followed by Tukey multiple comparison tests. * and # denote significant difference from N and RH groups, respectively. N: normal; RH: rheumatoid arthritis; ISN26 Placebo: unloaded optimized formulation; ISN26: optimized formulation; CCP: cyclic citrullinated peptide; MCP-1: monocyte chemoattractant protein-1.

As depicted in Figure 6, CFA-induced RA resulted in pronounced elevation in the serum levels of a) anti-CCP and b) MCP-1. Elevation of these biomarkers during RA is reported by many investigators (Zhang et al., 2019). On the contrary, once a week IA administration of the optimized formulation of piroxicam was accompanied by marked reduction in their levels by almost 54% and 73%, respectively. These findings may be explained due to the previously reported efficacy of piroxicam as an anti-inflammatory drug. Saini et. al. reported the significant drop in the expression of both anti-CCP and MCP-1 upon treatment with piroxicam (Saini et al., 2013). Moreover, the treatment with the drug free optimized formulation alone showed significant decrease in the level of MCP-1 only.

##### 4.3.5.3 Effect of optimized piroxicam formulation on relative protein expression of STAT-3 and RANKL

**Figure 7.**
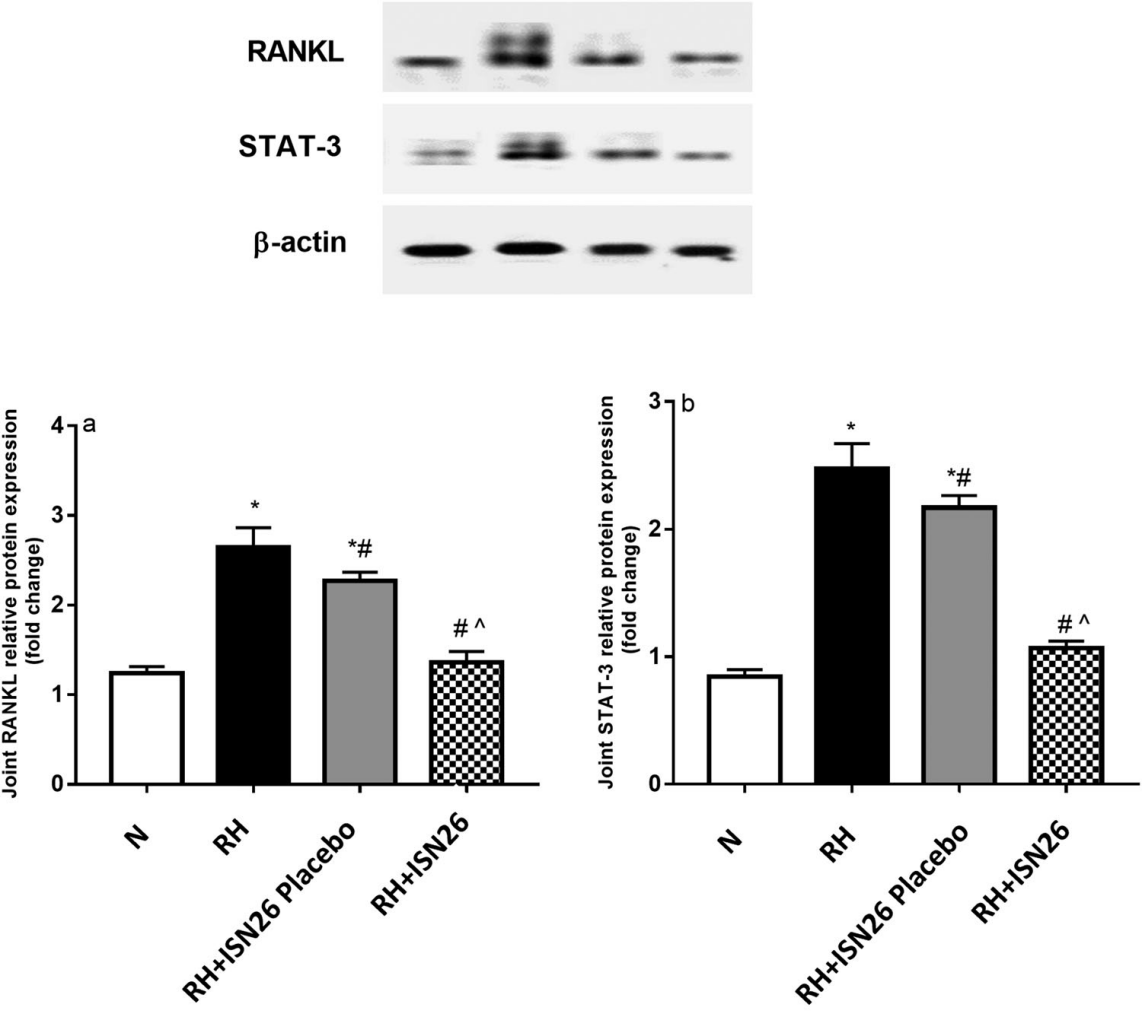
Effect of intra-articular optimized piroxicam formulation on the joint relative protein expression of (a) RANKL and (b) STAT-3 in CFA-induced arthritis in rats. Data are expressed as mean ± S.D. Statistical analysis was done using one-way ANOVA followed by Tukey multiple comparison tests. *, # and ^^^denote significant difference from N, RH and RH + F groups, respectively. N: normal; RH: rheumatoid arthritis; ISN26 Placebo: unloaded optimized formulation; ISN26: optimized formulation; RANKL: receptor activator of nuclear factor kappa-Β ligand; STAT-3: signal transducer and activator of transcription-3.

As illustrated in Figure 7, rats with RA showed significant increase in the relative protein expression of a) RANKL and b) STAT-3 in the joints as reported previously by our team (Kamel et al., 2019, 2018).On the contrary, administration of optimized piroxicam formulation resulted in profound reduction in their relative protein expression as compared to diseased group. In quite consistent manner, the selective COX-II inhibitor, celecoxib was found to suppress arthritis-related increase in bone resorption and diminish osteoclast progression within bone marrow via downregulation of RANKL/osteoprotegerin ratio and IL-6 mRNA expression in inflammed synovial tissue (Taketa et al., 2008). Moreover, piroxicam was reported to protect against inflammation by inhibiting proinflammatory cytokines and STAT3 signaling (Saini et al., 2013).

##### 4.3.5.4 Effect of optimized piroxicam formulation on the contents of IL-17, MMP-3, NF-κB and TNF-α

**Figure 8.**
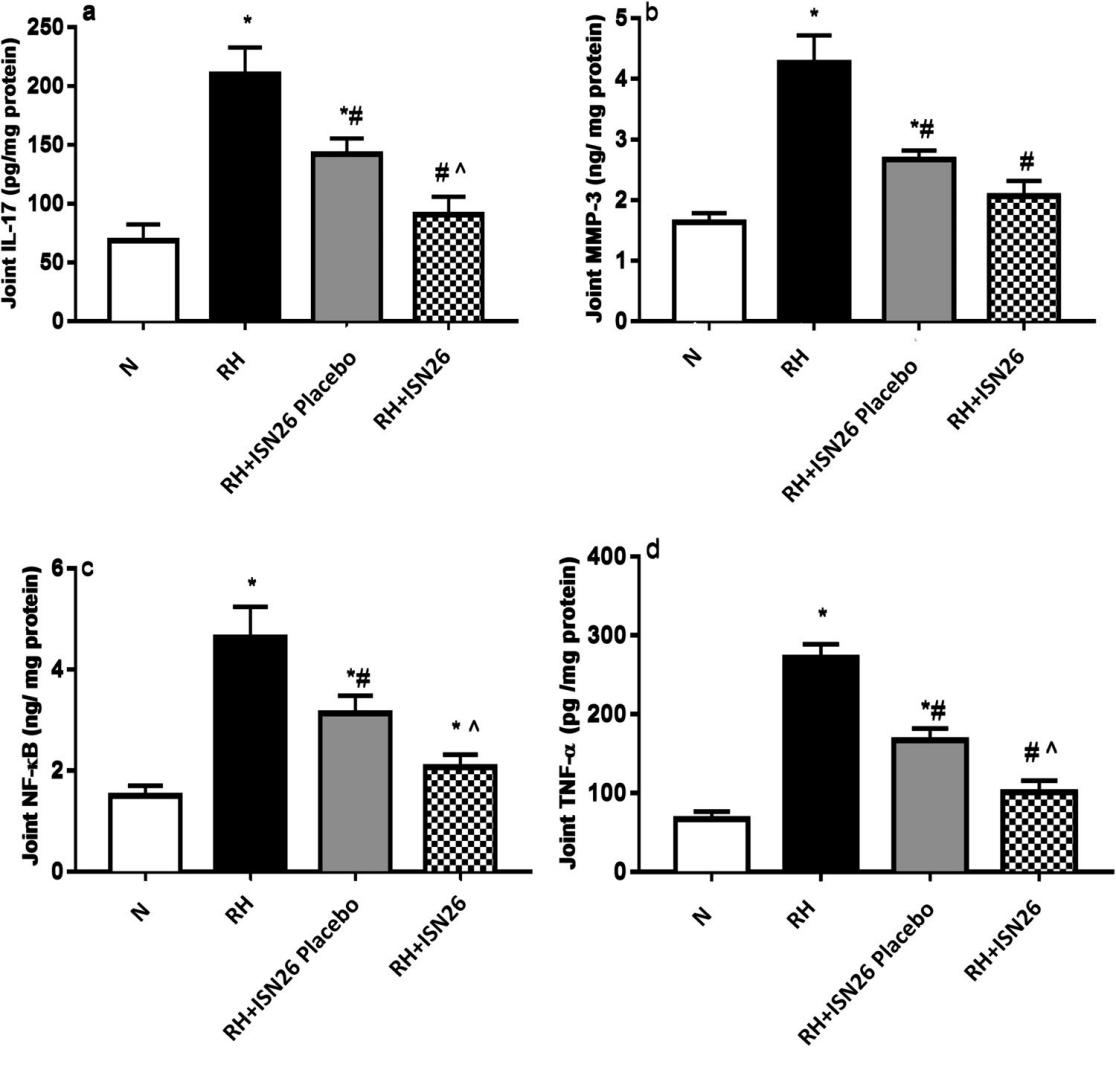
Effect of intra-articular optimized piroxicam formulation on the joint contents of (a) IL-17, (b) MMP-3, (c) NF-κB and (d) TNF-α in CFA-induced arthritis in rats. Data are expressed as mean ± SD. Statistical analysis was done using one-way ANOVA followed by Tukey multiple comparison tests. *, # and ^^^denote significant difference from N, RH and RH + F groups, respectively. N: normal; RH: rheumatoid arthritis; F: optimized formulation; PIRO: piroxicam; IL-17: interleukin-17; MMP-3: matrix metalloproteinase-3; NF-κB: nuclear factor kappa-B; TNF-α: tumor necrosis factor-α.

CFA-induced arthritis was further accompanied by significant inflammatory joint reaction as shown in Figure 8. Marked rise in the contents of a) IL-17, b) MMP-3, c) NF-κB and d) TNF-α was noticed. On the other hand, once a week treatment with the optimized piroxicam formulation markedly hampered their contents. In support, piroxicam significantly suppressed mRNA expression levels of TNF-α, IL-17 and NF-кB (Shabbir et al., 2016).

## 4. Conclusion

An artificial intelligence approach was adopted for the design, characterization and optimization of surface targeted ISNs for the IA delivery of piroxicam through pursuing a full factorial experimental design. The optimized formulation presented suitable rheometric characteristics with proven ease of injectability for application as an IA injection. On the other hand, the analysis of TEM images corroborated that the ISNs maintained a spherical dense shape with minor aggregations*. In vitro* release studies showed the sustained release profile of the optimum formulation confirmed with the biological investigation of its efficacy. A comparative study was conducted to compare the efficacy of the IA optimum formulation (ISN-26), its drug-free analogue (placebo), oral drug solution and IA drug suspension. In addition, the optimized formulation restored joints histological architecture as evidenced by the accelerated chondrogenic maturity and significant restoration of articular cartilage thickness accompanied by marked anti-inflammatory potential related to repressing the pivotal chemokines and minimizing the primary protein expression of STAT-3 and RANKL. Such findings revealed the potentiality of the innovated ISNs for effective IA delivery of piroxicam for RA treatment based on weekly administration regimen.
